# Mediterranean Diet Adherence, Body Composition and Performance in Beach Handball Players: A Cross Sectional Study

**DOI:** 10.3390/ijerph18062837

**Published:** 2021-03-10

**Authors:** Alejandro Martínez-Rodríguez, María Martínez-Olcina, María Hernández-García, Jacobo Á. Rubio-Arias, Javier Sánchez-Sánchez, Daniel Lara-Cobos, Manuel Vicente-Martínez, Maria José Carvalho, Juan Antonio Sánchez-Sáez

**Affiliations:** 1 Department of Analytical Chemistry, Nutrition and Food Science, Alicante University, 03690 Alicante, Spain; maria.martinezolcina@ua.es (M.M.-O.); mhg30@alu.ua.es (M.H.-G.); 2 Alicante Institute for Health and Biomedical Research (ISABIAL Foundation), 03010 Alicante, Spain; 3 Department of Education, University of Almeria, 04120 Cañada, Spain; jacobo.rubio2@gmail.com or; 4 School of Sport Sciences, Universidad Europea de Madrid, 28670 Madrid, Spain; javier.sanchez2@universidadeuropea.es; 5 Italian Handball Federation, Stadio Olimpico (Curva Nord), 00135 Roma, Italy; daniel.lara.cobos@gmail.com; 6 Faculty of Health Science, Miguel de Cervantes European University, 47012 Valladolid, Spain; mvmartinez11006@alumnos.uemc.es; 7 Center of Research, Education, Innovation and Intervention in Sport (CIFI2D), Faculty of Sports, University of Porto, 4200-450 Porto, Portugal; mjc@fade.up.pt; 8 GDOT Research Group, Faculty of Sport, Universidad Católica San Antonio de Murcia, 30107 Murcia, Spain; jasanchez419@ucam.edu

**Keywords:** nutritional habits, beach handball players, bioimpedance, anthropometric profile, team sports

## Abstract

Background: Beach handball (BH) is a sport in which sporting performance is influenced, together with team interaction, by individual performance in terms of strength. Body composition is one of the main factors for sports performance and eating habits can condition this variable. The Mediterranean diet (MD) can significantly reduce the risk of mortality or cardiovascular disease. In addition, the Spanish Ministry of Agriculture, Food and Environment carries out different campaigns to promote it among young athletes, establishing it as a suitable diet for sports. Objectives: The main aims of the study are to assess body composition, physical activity and adherence to the MD of beach handball players. It also aims to evaluate age group differences in male and female players, as well as studying the possible relationship between MD, body composition and performance variables. Methods: A total of 59 Spanish BH players were recruited in the national championship of BH in the province of Alicante. Thirty-eight male (14 junior; 17.0 ± 0.1 years and 24 seniors; 25.5 ± 4.7 years) and twenty-one female (7 junior; 16.1 ± 1.46 years and 14 seniors; 23.2 ± 2.0 years) BH players participated in this study. The questionnaire to evaluate eating habits was Mediterranean diet adherence (KIDMED). Body composition was measured with electrical bioimpedance. Strength was evaluated by means of a maximum isometric handgrip test of the dominant hand with handgrip and height of jump by counter-jump on contact platform. In the statistical analysis, descriptions and correlations between the study variables were made. Results: In females, when the adherence to the MD is lower, the weight is higher, the lean body mass is lower and worse results are observed in performance tests. As for males, there are differences in weight and lean body mass according to category. Conclusions: Adequate eating habits are related to the weight of beach handball athletes. In addition, specifically with junior players, it has been observed that adherence to the MD correlates with weight.

## 1. Introduction

Beach handball (BH) is a relatively new type of team sports derived from team handball [1]. It is an adaptation of team handball played on sand, basically on the beach. The sand surface enables players to perform acrobatic moves. It was created in 1992 in Italy and has experienced rapid growth. The International Handball Federation (IHF) absorbed this modality and held the first world championship 12 years later [2]. Currently, there are national circuits (Germany, Spain, Poland or Portugal), Continental Championships or World Championships. BH is a sport with more offensive than defensive characteristics and it works with a constant numerical superiority in the attack. The positions are: front (wings/specialists), back (pivots/defenders) players and goalkeepers [3]. BH is a heterogeneous high-intensity sport with a variety of physiological demands involving short periods of maximal power and strength, with short sprints, jumps and throws throughout a match [4].

BH, like handball, is an intermittent exercise that mainly uses aerobic metabolism, interspersed with high-intensity actions that greatly tax anaerobic metabolism and in which neuromuscular fatigue may occur during the games [5]. The time between each change of intensity and the number of intense actions and movements suggest a high anaerobic energy turnover during critical periods of a game.

BH matches are played with three players on the line and a goalkeeper (defense). The game rules encourage a tactical situation in which, during attacks, a reserve player typically changes places with the goalkeeper. Moreover, one or two players typically act only in defense and are replaced as soon as the team gains possession of the ball. Thus, the game is always played with eight players (four per team) who are constantly entering and leaving the game [6].

The dehydration and hyperthermia, to which BH players are exposed due to their practice in hot environments, are important physiological stress factors that can seriously hamper overall physiological function, and cognitive and athletic performance. They are also risks that pose challenges to the recovery process [7,8,9,10]. Recovery during the season involves a systematic approach to maintaining the physical and mental readiness of athletes to perform in the next competition. Multiple variables can influence and improve recovery; the principal one, nutrition, promotes muscle regeneration [11], glycogen restoration [12] reduces fatigue, and supports physical and immune health.

The American Dietetic Association, Dietitians of Canada and the American College of Sports Medicine emphasize the importance of diet and how sports performance and exercise recovery are reinforced by optimal nutrition [13,14]. These organizations recommend appropriate food and fluid selection, and consider timing and supplementation options, since athletes need to consume adequate energy during periods of high-intensity and/or long-term training to maintain body weight, health and maximize training effects [13,14]. Among the different types of diets recommended, the Mediterranean diet (MD) is the one that has the most positive effects on health [15,16,17,18].

The Mediterranean diet (MD) is characterized by high consumption of whole grains, legumes, fresh fruits, vegetables and olive oil, moderate to high consumption of fish, moderate consumption of dairy products and low consumption of red meat products. It has been accepted as one of the healthiest dietary patterns in the world [19]. There is increasing evidence that health-related behaviors are grouped [20]. For instance, combining healthy eating habits and regular physical activity is effective in maintaining and improving health, as well as better mental and physical well-being [19].

Moreover, to succeed in sports, it is usually important to have specific body attributes. Body composition (BC) plays a critical role in athlete health and sport performance. Extreme levels of fat mass (FM) may bring about severe health consequences. Generally, smaller amounts of FM coupled with greater amounts of muscle mass (MM) are favorable for athletes [21] and provide the basic foundation for sport-specific technical skills and locomotor activities [22]. The specific balance of FM and lean body mass (LBM) may be dependent upon sport position [21,23,24].

Among anthropometric features, hands and wrists are key factors in reaching the highest speeds in overarm throws. For this reason, some studies have focused on the relationship between specific hand measures and different throwing tests or handgrip strength [25,26]. Massuca et al. [27], for example, observed that some fitness results like countermovement jump (CMJ) height and average power can be useful to identify talent or assess performance [28].

Despite the growth experienced in BH at an organizational and participation level in recent years, data regarding the characteristics of the game, the BC, physical characteristics and eating habits of athletes are scarce [1,29,30]. Obtaining accurate data about the anthropometric and physical profiles is necessary to increase knowledge relating to this sport and identify the most important qualities for successful performance.

In accordance, the objectives of this study were to assess body composition, physical activity, and adherence to MD of beach handball players. It also aims to evaluate age group differences (senior vs junior) in male and female players. As well as studying the possible relationship between MD, body composition and performance variables (isometric handgrip strength and countermovement jump).

## 2. Materials and Methods

### 2.1. Study Design

A descriptive and cross-sectional design was used to analyze the influence of sex and category on the BC, performance, adherence to the MD and physical fitness in elite beach handball players. Deontological standards recognized by the Declaration of Helsinki (revision of Hong Kong in September 1989 and Edinburgh in 2000) were followed, and the research was carried out in accordance with the recommendations for Good Clinical Practice of the EEC (document 111/3976/88 of July 1990). The Institutional Ethics Committee of the Catholic University of San Antonio (Murcia) approved the study and permission was requested from the RFEBM to publish the data collected at the Concentration.

### 2.2. Participants

A total of 59 BH players from the Spanish National Beach Handball Team were recruited at the Arena Handball Tour® (Spanish National Circuit of BH) held in Orihuela, Alicante (a region of south-east Spain). Thirty-eight male BH players 14 junior (17.0 ± 0.1 years; 15–18 years, 176.9 ± 6.7 cm and 70.1 ± 11.2 kg) and 24 seniors (25.5 ± 4.7 years; 18–35 years, 183.0 ± 6.4 cm and 81.3 ± 7.6 kg) and 21 female BH players (seven junior—16.1 ± 1.46 years; 14–17 years, 165.0 ± 9.9 cm and 56.3 ± 8.7 kg) and 14 seniors (23.2 ± 2.9 years; 18–28 years, 166.0 ± 6.3 cm and 63.7 ± 8.9 kg) participated in this study. The youngest players were 14 years old. Participants were informed about the study objective and gave their written consent to participate. In the case of participants who were underage, the consent of their parents or legal guardians was obtained. Anonymity was preserved for all participants.

### 2.3. Data Collection

#### 2.3.1. Assessment of Mediterranean Diet Adherence

Because most of the sample were adolescents, the KIDMED questionnaire was used to evaluate the adherence to a Mediterranean diet during a face-to-face interview between the patient and a certified nutritionist. It consists of 16 items, where there are 4 questions denoting a negative connotation to the Mediterranean diet (consumption of fast food, baked goods, sweets, and skipping breakfast) and 12 questions denoting a positive connotation (consumption of oil, fish, fruits, vegetables, cereals, nuts, pulses, pasta or rice. dairy products, and yoghurt). Questions denoting negative connotation are scored with −1. while positive connotation questions are scored with +1. According to the KIDMED index, a score of 0–3 reflects poor adherence to the Mediterranean diet, a score of 4–7 describes average adherence, and a score of 8–12 good adherence [31].

#### 2.3.2. Body Composition

Body composition measurements were performed on the BH players on the first day of the championship before the matches. Body composition parameters included height (cm), weight (kg), body mass index—BMI (kg/m^2^), water (%), LBM (kg) and bone mass (kg). First, height was determined using a mobile stadiometer (Seca 213, SECA Deutschland, Hamburg, Germany) to the nearest millimeter, with the participant’s head maintained in the Frankfort horizontal plane position. Each participant took an inhalation at the moment of measurement, while remaining standing with the heels, buttocks and back in contact with the height meter (cm), following the recommendations of the Spanish group of Kinanthropometry [32]. The remaining variables were measured with bioelectric impedance analysis in all subjects (Tanita BC-418, Tokyo, Japan) as it is a non-invasive, low cost and commonly used approach for BC measurements and assessment of clinical condition [33]. Analysis of bioimpedance was obtained at more than two frequencies, therefore, multiple frequency bioimpedance analysis (MF-BIA) was used. MF-BIA is based on the finding that the fluctuating percentages of extra cellular fluid and total body water can be assessed by exposing it to low and high frequency electric currents, respectively [34]. It has been observed that Tanita BIA machines appear to be both reliable and valid for predicting the fat-free mass (FFM) of male and female college students. Therefore, it is also appropriate to use for body composition assessment in adults [35]. In addition, this method has been previously used for the assessment of BC in athletes [36,37,38]. It was observed that BIA, and many of the anthropometric equations used to estimate LBM change, showed high correlations with dual-energy X-ray absorptiometry (DXA) data [38]. The reliability of BIA measurements is influenced by various factors related to the instrument itself, including electrodes, operator, subject and environment. BIA assumptions beyond its use for body composition of the human body are empirically composed of cylinders; LBM contains virtually all the water and conducting electrolytes in the body, and its hydration is constant [39]. Correlations, bias, limits of agreement and systematic, standardized, and qualitative differences between DXA %BF and BIA showed moderate to large positive correlations (r from 0.44 to 0.58; all p < 0.05) [40]. Furthermore, it has been found that there is sufficient correlation (*r*-values) between Tanita and the four-compartment model for body fat measurement [41]. All players were measured before the start of the championship in a postprandial state, in light clothing, barefoot and in standing position, since the errors that may arise from a more practical standing measurement rather than lying are minimal [42].

#### 2.3.3. Handgrip Strength

Isometric handgrip strength (HGS) was measured twice for the dominant hand using a calibrated handgrip dynamometer (Takei 5101, Tokyo, Japan) with 30 s of passive recovery between trials. Participants sat with 0° of shoulder flexion and elbow flexion, and the forearm and hand in a neutral position and exerted their maximal strength for 5 s [43]. The highest value of the dominant hand was recorded and used for statistical analysis as the maximum voluntary handgrip strength.

#### 2.3.4. Countermovement Jump

The countermovement jump (CMJ) was determined using a force platform (Quattro Jump, version 1.04; Kistler Instrument AG, Winterthur, Switzerland). The players began from the upright position (0 degrees knee angle), making a downward movement to 90° of knee flexion and simultaneously beginning the push-off into full extension of the legs. The BH players performed three jumps and the highest one was included for statistical analysis [1,44]. Jump height, maximal force before take-off and the mean power were evaluated in this test. This test offers a non-invasive assessment that can be performed in a time efficient manner, making it an attractive field measure to evaluate neuromuscular performance [45].

#### 2.3.5. Statistical Analyses

For the data analysis, the programs Jamovi 1.1.3.0 and SPSS IBM Statistics version 24 (Chicago, IL, USA) were used. For descriptive statistics (mean ± standard deviation) and inferential analysis, the Kolmogorov–Smirnov test was conducted to find the normality of the data, followed by Levene’s test to determine the homogeneity (*p* > 0.05). Analysis of covariance (ANCOVA) with the correction of Bonferroni was used to compare differences between age groups (junior vs. senior), controlling for the effect of BMI. Additionally, Eta-squared (η^2^p) was calculated with <0.25, 0.26–0.63 and >0.63, considered small, medium and large effect sizes, respectively. The correlations between height (cm), body mass (kg), BMI (kg/m^2^), fat mass (%), LBM (kg), HGS (kg), CMJ (cm) and KIDMED score were determined using Pearson’s product-moment correlation coefficient (*r*), with 95% confidence intervals (CI). To interpret the results, the threshold values for the Pearson product-moment used by Salaj and Markovic [46] were used: low (*r* ≤ 0.3), moderate (0.3 > *r* ≤ 0.7) to high (*r* > 0.7). Statistical significance was set at *p* < 0.05.

## 3. Results

A total of 59 BH players of Spanish nationality participated, of which 38.1% were in the senior category and 61.90% in the junior category, with a mean age of 17.0 ± 0.1 and 25.5 ± 4.7 years, respectively. Basic descriptive statistics of the total sample in the KIDMED questionnaire are presented in Table 1 and Table 2.

Of all females, in juniors the highest prevalence was in these following groups: likes pulses and eats them >1/week +1 (33.33%), use of olive oil at home (33.33%) and consumption of dairy products for breakfast (28.57%). However, in the case of seniors, in addition to the consumption of legumes (57.14%) and olive oil (66.673%), a high percentage of the sample indicated that they ate fruits and vegetables every day (57.14%). As for the scores obtained by men, for juniors the items with the highest percentage of compliance are use of olive oil (34.21%), consumption of fish 2–3 days/week (31.58%) and two yoghurts or 40g cheese daily. However, a considerable number of this team, 10 out of 14, go to a fast-food restaurant more than one day a week. They do not eat fruit or juice on a daily basis, and they also consume commercially baked goods or pastries for breakfast. Finally, in the case of senior men, the use of olive oil (60.53%), the consumption of legumes (47.37%) and the consumption of fruits and vegetables daily (47.37%) were the items with the highest percentage scores. In addition. they consume pasta or rice almost daily (≥5 days/week) and cereals for breakfast; however, they do not consume more than one serving of vegetables per day and for breakfast they use commercially baked goods or pastries.

Looking at the KIDMED index score (Table 2), the majority of the population has a moderate adherence to the Mediterranean diet: 76% of total females and 66% of males. Only 9.52% of females and 20% of males have low adherence.

Table 3 shows the mean and standard deviation values for the body composition, sport performance and MD adherence of female BH players. There were no statistically significant differences between junior and senior players in any of the variables analyzed (*p* > 0.05).

Table 4 shows mean and standard deviation values for the BC, sport performance and MD adherence of male BH players. Statistically significant differences (*p* ≤ 0.05) were found between seniors and juniors in height, weight and LBM. Weight, height and LBM are higher in the senior group than in the junior group.

The score observed in the KIDMED was not different for junior and senior females (dif. mean = −1.64 ± 1.30; Effect size—ES = 0.08, *p* = 0.225). In males, the scores were slightly lower, with the difference between senior and junior being less (dif. mean = −0.315 ± 0.94; ES = 0.016, *p* = 0.739).

Statistically significant correlations (*p* < 0.05) are shown in Table 5 for junior females between height and LBM. Positive correlations between height and LBM (CI 95% = 0.469 to 0.979), between HGS and height (CI 95% = 0.461 to 0.978) and between HGS and CMJ (CI 95% = 0.111 to 0.953) were observed for junior female players. However, there were also significant negative correlations between KIDMED scores and body mass (CI 95% = −0.981 to −0.506), and between KIDMED scores and HGS (CI 95% = −0.971 to −0.346).

Regarding males, statistically significant correlations (*p* < 0.001) are shown in Table 6 between BMI and FM (CI 95% = 0.217 to 0.807) in the senior category. A higher BMI correlated with a greater %FM. In the junior category, statistically significant correlations (*p* < 0.05) were observed both between BMI and body mass (CI 95% = 0.769 to 0.971), FM and body mass (CI 95% = 0.425 to 0.912; *p* < 0.001), between HGS and LBM (CI 95% = 0.190 to 0.201; *p* < 0.001) and between HGS and body mass (CI 95% = 0.024 to 0.805).

## 4. Discussion

BH is a relatively new sport, which means that so far it has received little intensive investigation, so available data are scarce [1]. The main objective of this study was to determine the body composition, the sport performance (through handgrip and CMJ) and the adherence to the MD. It also aims to see if there is any difference between categories of males and females in terms of each variable. Positive correlations were obtained between different body composition variables and performance tests. Regarding adherence to the MD and some characteristics of both body composition and physical qualities, the correlation was negative. In general, the diet should be improved, as they present a moderate adherence to the MD. To our knowledge, only one study has been published on the physiological and nutritional profile of HB players [29] and another analysed the body composition profile and performance in females [1].

Assessment of body composition in athletes may help to optimize competitive performance and monitor the success of training regimens and thus is of considerable interest to sports professionals. It has been stated that improved body composition in athletes is associated with enhancements in cardiorespiratory fitness and strength [47]. The whole-body level of body composition characterizes body size and configuration, which is often described by anthropometric measures such as body weight, skinfold thicknesses, circumferences, and body mass index (BMI) among others [48]. The mean data for height in senior females, were similar to the data found in the study carried out by Becerra’s et al. [1] and by Silva et al. [29]. In the present study, the junior and senior female players had a similar weight to those obtained in the study conducted by Becerra et al. [1] and those of Silva et al. [29]. However, it appears that the players in our study had a slightly higher % FM compared to the players of Becerra et al. [1] research. Nevertheless, the players in Silva’s et al. [29] study presented higher results. From the study conducted by Campa et al. [49] that aimed to establish reference values of body composition through BIA in male and female athletes, the results of athletes that were classified as team sports (basketball, field hockey, handball, rugby, soccer, volleyball and water polo) were selected. The results were very similar, especially in the senior population, this makes sense since the age is similar. In addition to weight, height and fat-free mass are also similar. However, the male and female players in this study had lower fat values. This is to be expected, since the study population is elite. In another study carried out in an athlete population [47], they also tried to establish reference values, however, they used DXA and reported that the DXA reference values presented in that study are only comparable with those derived by use of the Hologic fan beam DXA scanner (software version 12.4 or higher).

In terms of performance tests, handgrip and CMJ, junior players in this study had lower scores, while the senior players had slightly higher scores. As observed in a study of volleyball players [50], this observation may be because these ‘specialized improvements’ might be directly related to the neuromechanical adaptations provoked by sport-specific actions that are repetitively performed during training and matches. For this reason, higher scores were observed in those senior players who have been training longer. As proper jumping is essential in BH, it is rational to assume that athletes who are able to jump higher may be ‘naturally selected’ over the younger categories, which could limit the progressive development of this ability throughout the age categories [51,52]. The scores were 30.1 ± 5.9 centimeters (cm) and 39.6 ± 25.5 cm for juniors and seniors, respectively, compared to 34.92 ± 5.38 cm [1]. This makes sense, since the female players in Becerra’s et al. [1] study had a similar age to seniors.

On the handgrip test, the average score was 29.0 ± 5.4 kg for juniors and 35.1 ± 7.8 kg for seniors, compared with those of Becerra’s et al. [1], which were 4.35 ± 4.08 kg. The estimation of handgrip strength is of immense importance in sports like wrestling, tennis, football, handball, basketball, volleyball and baseball where a sufficient degree of grip strength is necessary to be successful. Handgrip strength is a physiological variable that is affected by a number of factors including age, gender and body size [53]. In the same line as previously observed, right/left handgrip strength was positively correlated with weight and height [53,54,55].

As far as male players are concerned, no studies have been carried out analysing either BC or performance tests in BH, so there is no scientific evidence to compare with. On the contrary, there are many investigations with handball players who play on a court. Both Bojan Masanovic et al. in 2018 [56] and Souhail Hermassi et al. in 2019 [44] measured the body composition characteristics of handball players in their research. In junior male players, the mean data for height, weight and % FM were higher in handball players [44,56] than in BH players. However, the players in our study showed higher values of CMJ; than players in the same category as those in the Hermanssi et al. study [44]. The CMJ has been associated with slow stretch-shortening cycle (SSC) (>250 m in duration) performance, which has been related to sprint acceleration, where ground contact time is longer [57]. This makes the CMJ assessment a fundamental tool in appraising key performance indices among BH athletes, who require frequent acceleration and deceleration [58]. 

The body mass index (BMI; weight/height^2^) is a parameter that is widely used in adult populations as an internationally recognised indicator of being overweight and obese. In the case of underage children International Obesity Task Force criteria linking adult cutoff points of overweight/obesity (25 kg/m^2^ and 30 kg/m^2^, respectively) to BMI centiles for children and adolescents for defining underweight, normal, and overweight were used [59]. Fortunately, the BMI of all four groups (males; junior, senior and females; junior, senior) rests in the area of normal weight according to the established literature and did not show any significant differences between groups. However, it is known that the BMI is not a useful tool for determining the ideal weight in an athlete, since it is based on the assumption that all the weight that exceeds the values determined by the height–weight tables will correspond to FM. It is clear that being overweight may correspond to the increase in MM and/or bone mass [60]. Its correlation with body fat is relatively poor, given that it shows little sensitivity when determining the different deposits of fat, mainly abdominal, due to its extensive relationship with noncommunicable diseases like obesity [61]. In order to correct this aspect, the BMI was complemented with the body fat percentage.

In general, it has been shown that adherence to the MD is associated with a reduction in different factors associated with obesity, in addition to high levels of quality of life and better physical and mental health [19,31]. In general, both females and males in the present study stated that they did not eat more than one fresh or cooked vegetable. However, in the case of men, both juniors and seniors, they confirmed that they did not eat fruits either fresh or cooked. As for regular nut consumption (at least 2–3/week), all teams had a higher proportion of players who did comply with it, except for the junior women. In addition, of the total sample, 38% consume dairy products for breakfast, which is an aspect that needs to be improved. In a study performed with 1717 European adolescents (N = 900 boys, N = 817 girls) relating adherence to the MD and body composition, it was observed that not eating rice or pasta almost daily (≥5 times per week) increases the risk of suffering from over waist circumference, overfat and overweight [62]. Of the present study, approximately half did not meet this item (47.45% of the total sample). In turn, the non-over waist circumference participants had a higher regular fish consumption (2–3 times per week), hence not reaching that consumption rate being a risk factor and more so for a second serving of fruit daily, whose non-consumption increases the relative risk of over waist circumference. Non-overweight participants also showed a superior regular nut consumption (≥2–3 times per week), thus not reaching such consumption increases the risk of overweight. In a different study [63], they observed how ideal adherence to the MD appears to be associated with elevated levels of cardiorespiratory fitness. Similarly, participants with high adherence to the MD had better parameters of respiratory capacity due to a better body composition and cardiorespiratory profile.

The bioactive components best recognized as responsible for the beneficial effects of this diet are antioxidants, fiber and phytosterols, from vegetable products, vegetables, fruits, legumes and virgin olive oil; monounsaturated fatty acids present in olive oil; omega-3 fatty acids from marine products and nuts; and probiotics derived from fermented foods such as cheese and yogurt, among others [64]. Specifically, omega-3 fatty acids could bring various benefits to athletes by attenuating the generation of oxidative stress and, thus, improving muscular performance and immune function [65]. As for probiotics, there are several different results [66] however In a study investigating the effect of a multi-strain probiotic yogurt on performance in adolescent female endurance swimmers over 8 weeks, there was a significant improvement in maximum oxygen volume (VO2 max) [57]. Multiple mechanisms describe how fiber influences satiation and satiety contributing to the control of body weight [67].

In the present study, it was observed that both females and males showed moderate adherence to the MD. Specifically, the teams with the highest scores for their athletes were the senior men. The majority of the sample (69%) obtained scores between 4 and 7, therefore, moderate adherence. This is in the same line as other studies, as it has been observed that, despite the health benefits that it presents, adherence to the MD has been rapidly declining in Mediterranean countries, including Spain [68]. This fact agrees with the study carried out by Inge Spronk [69], in which also noted that elite female Australian athletes reported a better diet quality than male athletes. A previous systematic review [41] also reported that, regarding specific components of the MD, females were more likely than males to meet the recommendations for daily consumption of vegetables and red meat. Similar results were found by de Boer et al. [70] who concluded that differences could be driven by greater health consideration and awareness of climate change amongst females. This coincides with the results obtained, since it has been observed that 90.48% of females have an average or good adherence to the Mediterranean diet. While in the case of males it does not reach 80%, with a higher proportion of players with low adherence.

This is the first study to show a relationship between height and HGS, HGS and CMJ, and KIDMED scores with body weight and HGS in junior female players. As shown in Table 3, greater height means better results in the manual dynamometry test. Better results in the HGS also mean better results in the CMJ test. In terms of adherence to the MD, there was a negative correlation with both weight and HGS. This observation suggests that following a poor-quality eating pattern favors high body weight and lower strength in the upper body, specifically in hand strength.

For males, only the correlations between the HGS test, body weight and fat-free mass in junior players were significant. This suggests that the higher the body mass and fat-free mass, the better the results in the HGS test. The research on the determinants of BH performance from a physiological perspective has focused on the profiling of physical fitness characteristics of elite players. It is now well-established that elite handball players should have a high stature and body mass and high anaerobic power [71].

The present study has some limitations. It included a small number of players. Additionally, there are few studies to compare our results with. Finally, it should be considered that the questionnaire to evaluate adherence to the MD, KIDMED, is self-referential and does not report the amount of food ingested. In addition, due to the cross-sectional nature of this study, it is necessary to emphasize that a nutritional intervention based on the MD could influence sports performance and body composition. Additionally, the method of measuring body composition should be taken into account, as the use of bioelectrical impedance with the direct segmental technique in the standing position can lead to a gravitational effect on body fluids that causes some artifacts to occur in the estimated body composition measurements.

In future research, it would be interesting to study the relationship between adherence to the MD, taking into account quantities ingested, sporting performance, hours of physical activity, type of training carried out per week and the body composition characteristics of elite BH athletes. The study’s aim is to determine whether there are dietary deficiencies that influence performance, training quality and BC, and with this information, to draw up prevention and intervention programs with the objective of improving the athlete’s health and sporting career.

## 5. Conclusions

Female athletes which were studied from the Spanish BH team exhibited relatively high fat percentages compared to standards for international-level BH athletes. A high percentage of the sample regularly consumes olive oil at home; however, there is a higher proportion in the sample of those who do not consume vegetables and fruits on a daily basis than those who do. In addition, we also see that a high number of participants consume dairy products at breakfast. For junior female players, adequate eating habits, based on the MD, are related to weight. Moreover, there was a positive relationship between manual grip strength and muscular power in jumping. In conclusion, it seems that the greater the strength of the upper limbs, the greater the strength and power of the lower limbs. The grip strength of the dominant hand was positively correlated with height and fat-free mass in both male and female juniors. The BH players in this study had moderate adherence to the MD, and it would need to be improved. Finally, adequate adherence to the MD is not sufficient to obtain better results in performance tests. More studies are needed to be able to have reference data on this sport which is relatively new but has a great impact on society.

## Figures and Tables

**Table 1:** Mediterranean diet quality index statistics for total sample.

Study Variables	FEMALES								MALES							
	JUNIOR				SENIOR				JUNIOR				SENIOR			
	NO	(%)	YES	(%)	NO	(%)	YES	(%)	NO	(%)	YES	(%)	NO	(%)	YES	(%)
Fruit or fruit juice daily	2	(9.5)	5	(23.8)	2	(9.5)	12	(57.1)	8	(21.1)	6	(15.8)	6	(15.8)	18	(47.3)
Second serving of fruit daily	5	(23.8)	2	(9.5)	7	(33.3)	7	(33.3)	10	(26.3)	4	(10.5)	16	(42.1)	8	(21.1)
Fresh or cooked vegetables daily	3	(14.3)	4	(19.1)	7	(33.3)	7	(33.3)	9	(23.7)	5	(13.2)	12	(31.6)	12	(31.6)
Fresh or cooked vegetables > 1/day	7	(33.3)		-	9	(42.9)	5	(23.8)	11	(28.9)	3	(7.9)	18	(47.4)	6	(15.8)
Regular fish consumption (at least 2–3/week)	4	(19.1)	3	(14.3)	6	(28.6)	8	(38.1)	2	(5.3)	12	(31.6)	12	(31.6)	12	(31.6)
>1/week fast-food (hamburger) restaurant	5	(23.8)	2	(9.5)	8	(38.1)	6	(28.6)	4	(10.5)	10	(26.3)	10	(26.3)	14	(36.8)
Pulses > 1/week		-	7	(33.3)	2	(9.5)	12	(57.1)	6	(15.8)	8	(21.1)	6	(15.8)	18	(47.4)
Pasta or rice almost daily (≥5 days/week)	6	(28.6)	1	(4.8)	8	(38.1)	6	(28.6)	3	(7.9)	7	(18.4)	11	(28.9)	17	(44.7)
Cereal or cereal product for breakfast	4	(19.0)	3	(14.3)	6	(28.6)	8	(38.1)	5	(13.2)	8	(21.1)	9	(23.7)	16	(42.1)
Regular nut consumption (at least 2–3/week)	6	(28.6)	1	(4.8)	5	(23.8)	9	(42.9)	6	(15.8)	8	(21.1)	9	(23.7)	15	(39.5)
Use of olive oil at home		-	7	(33.3)		-	14	(66.7)	1	(2.6)	13	(34.2)	1	(2.6)	23	(60.5)
No breakfast	4	(19.0)	3	(14.3)	13	(61.9)	1	(4.8)	10	(26.3)	4	(10.5)	20	(52.6)	4	(10.5)
Dairy product for breakfast	1	(4.8)	6	(28.6)	5	(23.8)	9	(42.9)	5	(13.2)	9	(23.7)	10	(26.3)	14	(36.8)
Commercially baked goods or pastries for breakfast	5	(23.8)	2	(9.5)	11	(52.4)	3	(14.3)	10	(26.3)	4	(10.5)	18	(47.4)	6	(15.8)
Two yoghurts and/or 40 g cheese daily	2	(9.5)	5	(23.8)	8	(38.1)	6	(28.6)	4	(10.5)	10	(26.3)	10	(26.3)	14	(36.8)
Sweets and candy several times a day	5	(23.8)	2	(9.5)	9	(42.9)	5	(23.8)	6	(15.8)	8	(21.1)	12	(31.6)	12	(31.6)

% = percentage.

**Table 2:** Mediterranean diet quality total score for total sample.

KIDMED Index Score	N	(%)	N	(%)	N	(%)	N	(%)
Poor (≤3)	0	(0)	2	(9.5)	5	(13.2)	3	(7.9)
Average (4–7)	7	(33.3)	9	(42.9)	8	(21.1)	17	(44.7)
Good (≥8)	0	(0)	3	(14.3)	1	(2.6)	4	(10.5)

N = number of people; % = percentage.

**Table 3:** Female beach handball players’ descriptive and comparison (junior vs senior).

Study Variables	Female						ANCOVA Comparison (Adjusting for BMI)						
	Junior (*n* = 7)			Senior (*n* = 14)			Mean		SD	df	t	*p*-Value	η^2^p
**Age**	16.1	±	1.5	23.2	±	2.9							
**Body composition**													
Height (cm)	165.0	±	9.9	166.0	±	6.3	−1.26	±	4.01	18.0	−0.314	0.757	0.005
Weight (kg)	56.3	±	8.7	63.7	±	8.9	−0.357	±	2.42	18.0	−0.147	0.884	0.001
BMI (kg/m^2^)	20.6	±	2.1	23.3	±	3.2							
Fat mas (%)	15.2	±	4.9	18.6	±	6.8	1.55	±	1.65	18.0	0.940	0.360	0.047
Lean body mass (kg)	45.2	±	7.1	48.8	±	4.2	−1.1	±	2.43	18.0	−0.454	0.655	0.011
**Sport performance**													
Strength (handgrip)	29.0	±	5.4	35.1	±	7.8	−6.69	±	3.57	18.0	−1.87	0.077	0.163
Power explosive (CMJ)	30.1	±	5.9	39.6	±	2.5	−7.21	±	10.5	18.0	−0.688	0.500	0.026
**Mediterranean diet adherence**													
Total score (KIDMED)	5.6	±	1.9	6.15	±	3.2	−1.64	±	1.30	18.0	−1.26	0.225	0.08

SD: standard deviation; df: degrees of freedom; η^2^p: partial eta squared; BMI: body mass index.

**Table 4:** Male beach handball players descriptive and comparison (junior vs senior).

Study Variables	Male						ANCOVA Comparison (Adjusting by BMI)						
	Junior (*n* = 14)			Senior (*n* = 24)			Mean		SD	df	t	*p*-Value	η^2^p
**Age**	17.0	±	0.1	25.5	±	4.7							
**Body composition**													
Height (cm)	176.0	±	6.7	183	±	6.4	−7.34	±	2.26	35.0	−3.24	0.003	0.033
Weight (kg)	70.1	±	11.2	81.3	±	7.6	−6.24	±	1.81	35.0	−3.44	0.002	0.689
BMI (kg/m^2^)	22.5	±	3.7	24.3	±	1.8							
Fat mas (%)	8.7	±	5.7	11.6	±	5.2	−0.53	±	1.40	35.0	0.372	0.712	0.464
Lean body mass (kg)	60.2	±	7.4	68.2	±	6.6	−5.51	±	2.01	35.0	−2.74	0.01	0.315
**Sport performance**													
Strength (Handgrip)	46.9	±	6.9	51.6	±	9.2	−3.71	±	2.87	35.0	−1.29	0.204	0.037
Power explosive (CMJ)	43.7	±	14.6	40.4	±	13.6	−0.069	±	4.58	35.0	−0.015	0.988	0.136
**Mediterranean diet adherence**													
Total score (KIDMED)	5.2	±	2.8	5.7	±	2.6	−0.315	±	0.94	35.0	−0.335	0.739	0.016

SD: standard deviation; df: degrees of freedom; η^2^p: partial eta squared.

**Table 5:** Female beach handball players’ correlation between body composition, performance and MD adherence variables.

Study Variables		Junior							
	Female	Height (cm)	Body Mass (kg)	BMI (kg/m^2^)	Fat Mass (%)	LBM (kg)	HGS (kg)	CMJ (cm)	KIDMED Score
Senior	Height (cm)	n.a	0.827 *	0.242	−0.086	0.882 *	0.88 *	0.52	−0.874
	Body mass (kg)	0.255	n.a	0.744 *	0.26	0.928	0.653	0.348	−0.893 *
	BMI (kg/m^2^)	−0.153	0.902	n.a	0.543	0.558	0.114	0.023	−0.523
	Fat mass (%)	−0.128	0.818	0.936	n.a	−0.118	−0.264	−0.276	−0.082
	LBM (kg)	0.570*	0.813	0.531	0.34	n.a	0.777 *	0.471	−0.889
	HGS (kg)	−0.412	−0.242	−0.144	−0.328	−0.105	n.a	0.756 *	−0.845 **
	CMJ (cm)	0.035	0.202	0.131	0.161	0.167	−0.064	n.a	−0.692
	KIDMED score	0.147	−0.301	−0.396	−0.46	−0.047	−0.113	−0.608	n.a

LBM: lean body mass; HGS: handgrip strength; CMJ: counter movement jump (power explosive); n.a: not applicable; *: *p*-value < 0.05; **: *p*-value < 0.01. Grey color: values corresponding to senior category. White color: values corresponding to junior category.

**Table 6:** Male beach handball players’ correlation between body composition, performance and MD adherence variables.

Study Variables		Junior							
	Male	Height (cm)	Body Mass (kg)	BMI (kg/m^2^)	Fat mass (%)	LBM (kg)	HGS (kg)	CMJ (cm)	KIDMED Score
Senior	Height (cm)	n.a	0.181	−0.211	−0.089	0.361	0.414	0.348	−0.017
	Body mass (kg)	0.613 **	n.a	0.916 **	0.761 **	0.915 **	0.514 *	−0.329	−0.038
	BMI (kg/m^2^)	−0.16	0.678	n.a	0.793	0.750	0.309	−0.458	−0.042
	Fat mass (%)	−0.155	0.329	0.585 **	n.a	0.457	0.189	−0.449	−0.206
	LBM (kg)	0.707	0.793	0.314	−0.313	n.a	0.627 **	−0.229	0.137
	HGS (kg)	−0.103	0.03	0.117	−0.303	0.232	n.a	−0.143	0.315
	CMJ (cm)	0.224	−0.024	−0.276	−0.401	0.228	0.34	n.a	−0.494
	KIDMED score	−0.009	0.317	0.404	−0.119	0.403	−0.036	−0.162	n.a

LBM: lean body mass; HGS: handgrip strength; CMJ: counter movement jump (power explosive); n.a: not applicable; *: *p*-value < 0.05; **: *p*-value < 0.01. Grey color: values corresponding to senior category. White color: values corresponding to junior category.

## Data Availability

The data presented in this study is available on request from the corresponding author. The data are not publicly available due to is personal health information.
